# Role of Mitochondrial Pathways in Cell Apoptosis during He-Patic Ischemia/Reperfusion Injury

**DOI:** 10.3390/ijms23042357

**Published:** 2022-02-21

**Authors:** Sen Zhang, Sijing Rao, Meiwen Yang, Chen Ma, Fengfang Hong, Shulong Yang

**Affiliations:** 1 Experimental Center of Pathogen Biology, College of Medicine, Nanchang University, Nanchang 330006, China; zhangsen1628@163.com (S.Z.); raosijing379@163.com (S.R.); machen1008@163.com (C.M.); 2 Department of Physiology, College of Medicine, Nanchang University, Nanchang 330006, China; 3 Department of Surgery, Fuzhou Medical College, Nanchang University, Fuzhou 344099, China; 18379441123@139.com; 4 Department of Physiology, Fuzhou Medical College, Nanchang University, Fuzhou 344099, China

**Keywords:** mitochondria pathway, ischemia/reperfusion injury, apoptosis, liver

## Abstract

Hepatic ischemia-reperfusion injury is a major cause of post-operative hepatic dysfunction and liver failure after transplantation. Mitochondrial pathways can be either beneficial or detrimental to hepatic cell apoptosis during hepatic ischemia/reperfusion injury, depending on multiple factors. Hepatic ischemia/reperfusion injury may be induced by opened mitochondrial permeability transition pore, released apoptosis-related proteins, up-regulated B-cell *lymphoma-2* gene family proteins, unbalanced mitochondrial dynamics, and endoplasmic reticulum stress, which are integral parts of mitochondrial pathways. In this review, we discuss the role of mitochondrial pathways in apoptosis that account for the most deleterious effect of hepatic ischemia/reperfusion injury.

## 1. Introduction

Apoptosis is a normal physiological process of highly regulated cell death that occurs in most multicellular organisms [1]. Apoptosis plays an important role in the cell cycle and is an integral part of the immune system under physiological and pathological conditions [2]. Disorders of apoptosis are associated with autoimmune diseases, bacterial and viral diseases, heart disease, and neurodegeneration [3]. Apoptosis is defined as an energy-dependent cell death which is one of the pathological characteristics of ischemia/reperfusion injury (IRI) [4]. There are two different pathways of cell apoptosis, the extrinsic death receptor pathway and the intrinsic mitochondrial pathway. Irreversible intracellular genomic damage is caused by various stimuli including gamma-ray irradiation, endoplasmic reticulum stress, growth factor deprivation, and oxidative stress, which activates the intrinsic pathway, known as the mitochondrial pathway [5,6]. This pathway is responsible for mitochondrial electron transport chain breakage, reactive oxygen species (ROS) production, adenosine triphosphate (ATP) depletion, mitochondrial membrane potential (ΔΨm) decrease, and mitochondrial permeability transition pore (MPTP) opening, thus leading to cell apoptosis [7].

Mitochondria is one of the most pivotal places of energy metabolism and is most sensitive to ischemia and hypoxia. Some observe that mitochondria evolved a unique structure composed of two layers of membrane to preserve some basic functions of organelles [8]. The inner and outer membrane of mitochondria form a unique space within mitochondria, called the intermembrane space [9]. The inner membrane is highly impermeable and does not provide porin, but contains specific transport proteins. The mitochondrial outer membrane envelops the whole mitochondria and contacts the cytoplasm directly [10]. The outer membrane of mitochondria contains several intact proteins, known as porin or voltage-dependent anion-selective channel, which contains a channel permeable to <5000 Dalton molecules to transport macromolecules through mitochondrial membrane transfer proteins [11]. Under the action of various injury factors, the entry of apoptosis-related proteins between mitochondrial bilayer membranes into the cytoplasm begins, eventually leading to cell apoptosis. Ischemia and hypoxia usually firstly damage the structure and function of mitochondria in tissue cells [12]. Mitochondria play a key role in the apoptotic signal transduction pathway during cell apoptosis, which is manifested as electron transfer rupture of the mitochondrial respiratory chain, ROS production, ATP depletion, decreased mitochondrial membrane potential, MPTP opening, and even loss of release of outer membrane proteins, thus leading to cell apoptosis [7]. During liver ischemia reperfusion, mitochondrial structure and function are impaired and induce liver cell apoptosis, which are related to the opening of MPTP, the release of apoptosis-related proteins, the regulation of B *lymphocytoma-2* gene (Bcl-2) family proteins, mitochondrial dynamics imbalance, and endoplasmic reticulum (ER) stress [13,14,15].

There are two main pathways of apoptosis: exogenous death receptor pathway and endogenous mitochondrial apoptosis pathway. The external pathway refers to the death receptor pathway, activated by ligands and receptors. A variety of mediators, including TNFa, Fas ligand, TRAIL, and TLIA. The ligand TRAIL is activated by TNFa and other factors, and the pro-apoptotic mediators bind to their respective receptors to catalyze the activation of many Caspase8, which further leads to the activation and pro-apoptotic of Caspase3 [16,17]. The internal pathway is also known as the mitochondrial pathway. MPTP opens when the mitochondrial structure is damaged by external stimuli such as hypoxia, radiation, and cytotoxin. With the pro-apoptotic factors into the cytosol, the Bcl-2 family activates proapoptotic factors [2,18]. The translocation of Bax to the outer membrane of mitochondria causes changes in mitochondrial membrane permeability, then the mitochondrial transmembrane potential is to reduce and depolarize, releasing cytochrome-C and other active factors in the mitochondrial matrix. Cytochrome-C enters into the cytoplasm and binds to Apaf-1 to form the oligomer under the synergistic effect of ATP/dATP, which activates caspase-9 and downstream caspase-3, leading to cell apoptosis [19]. This review mainly discusses the role of mitochondrial apoptosis pathway in hepatic ischemia-reperfusion in recent years.

## 2. The Initiation of Apoptosis: Mitochondrial Fission and Fusion

**Figure 1:**
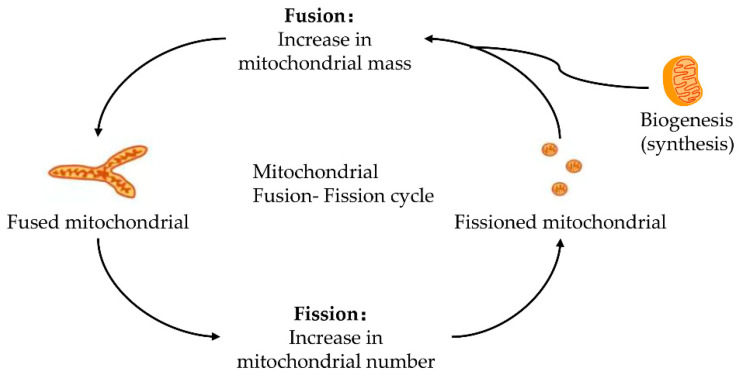
Fusion–fission cycle in mitochondria. Mitochondria strictly control the fusion and fission process through mitotic proteins embedded in the inner and outer membranes to maintain the dynamic balance of biological energy. The mitofusins and Opa1 mediate the fusion of the mitochondrial membrane. Fusion and fission belong to the mitochondrial quality control cycle. The growth and division of pre-existing mitochondria are also involved.

Mitochondria keeps its dynamic renewal by its continuous fission and fusion [20]. Mitochondrial fission causes its division, while its fusion leads to the binding and prolongation of phospholipid membranes in mitochondria (Figure 1). Mitochondria are strictly controlled by mitotic proteins embedded in the outer and inner membranes through stimulating mitochondrial fusion and fission. However, the dynamic cycle of fission and fusion in mitochondria is destroyed when under stress or damaged. The damaged dynamics in mitochondria may eventually lead to apoptosis by the excessive fission and reduced fusion in mitochondria after hepatic IRI [21].

### 2.1. Mitochondrial Fission and Fission

Mitochondrial fission is due to the increased mitochondrial division or decreased mitochondrial fusion [22,23,24]. The mitochondrial division damaged organelles are responsible for mitochondrial fission and mitosis. These fragmented organelles fuse to an interconnected network which renews the damaged mitochondrial DNA (mtDNA) [25]. Excessive fission causes mitochondrial breakage and activates its apoptosis pathway, thus aggravating tissue damage and cell apoptosis [26]. Additionally, excessive fission of mitochondria is a pre-symptom of cytochrome-C (cyt c) release [27], and the release of cyt c further promotes the mitosis of mitochondria.

Long et al. [21] showed that mitochondrial dynamics are related to the regulation of dynamin-related protein1(Drp1) and Mitochondrial fission protein 1 (MTFP1). Drp1 is a key protein in mitochondrial fission, which is mainly present in the cytoplasm and transferred to mitochondria after activation [28]. Hepatic IRI affects mitochondrial dynamics by translocating Drp1 to mitochondria in a fission-based manner [28]. During mitochondrial fission, the cytoplasm-localized Drp1 was recruited to its outer membrane which mediated membrane division. It is reported that interrupted mitochondrial fission by Drp1 protects hepatocytes from IRI-induced apoptosis [29]. In addition, Drp1-mediated mitochondrial fragmentation is regulated by Drp1 phosphorylation at Ser616 and Ser637, which results in the activation and inactivation of Drp1, respectively [28]. Drp1 phosphorylation at Ser616 promotes mitochondrial fission, and Drp1 phosphorylation at Ser637 seems to induce cell apoptosis. Drp1 Phosphorylation at Ser637 by protein kinase A (PKA) lengthens mitochondria to inhibit cell apoptosis, whereas Drp1 dephosphorylation at Ser637 by calcineurin (CaN) promotes mitochondrial fragmentation [30].

### 2.2. Mitochondrial Fusion

Mitochondrial fusion has long been regarded as a protective way to reduce mitochondrial fission [9]. During hepatic IRI, the fusion of damaged mitochondria could cause a beneficial effect to maintain the survival function of mitochondria [26] by preventing mitochondrial decomposition caused by the release of cyt c, xanthine oxidase (XO), and ROS in mitochondrial [31]. Fusion is associated with the redistribution of metabolites, proteins, and mtDNA in mitochondria. Fusion is also helpful for maintaining oxidative phosphorylation and integrity of mtDNA, and enhancing the synthesis of ATP [32].

The consequence of fusion is related to intimal proteins optic atrophy 1 (Opa1), mitochondrial fusion protein 1 (Mfn1), and Mfn2. Previous research has established that Opa1-induced fusion may be affected by extracellular regulated protein kinases (ERK) and sirtuin-3 (SIRT3) [33]. The highlight is that SIRT3 maintains mitochondrial homeostasis on IRI by enhancing mitochondrial fusion triggered by Opa1 [32]. Inhibition of ERK eliminates the regulatory effect of SIRT3 on Opa1 expression and mitochondrial fusion, resulting in mitochondrial damage and apoptosis of renal tubular epithelial cells [10].

## 3. “Switch” Role of Mitochondrial Permeability Transition Pore

**Figure 2:**
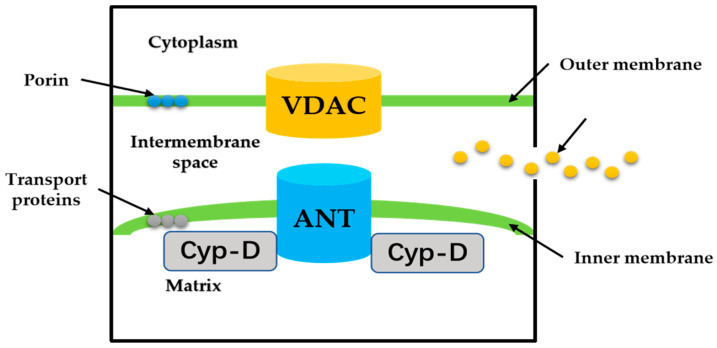
The proposed structure of the MPTP. Cyp-D is a small molecule in the mitochondrial matrix. VDAC on the OMM, ANT on the IMM.

MPTP is a group of protein complexes with non-specificity and voltage dependencies present between the inner and outer mitochondrial membranes (Figure 2). The outer membrane of mitochondria contains many intact proteins known as porin, which contains a channel that is permeable to <5000 Dalton molecules to transport macromolecules through mitochondrial membrane transfer proteins [11]. During mitochondrial damage, the inner membrane of the mitochondrial collapses once the mitochondrial permeability transition is initiated, allowing apoptosis-related proteins to rush out of the mitochondrial, and eventually leading to cell apoptosis [34,35].

The historical model of the mPTP comprised three components: voltage-dependent anion channel (VDAC) in the outer membrane of mitochondria, adenine nucleotide translocase (ANT) in the inner membrane of mitochondria, and cyclophilin D (CypD) in the mitochondrial matrix. Crompton et al. [36] demonstrated that CsA was an effective inhibitor of MPTP opening, and later studies showed that CsA inhibited pore opening through inhibition of matrix peptidyl prolyl cis-trans isomerase (PPIase). CsA is an inhibitor of CypD, which proves that the protein on MPTP is CypD [37]. Matrix PPIase activity promotes conformational changes in intimal proteins, but the composition of CypD is still under investigation. Studies have shown that liver mitochondria of CypD knockout mice are highly resistant to calcium-induced MPTP opening [38]. The mitochondrial phosphate carrier (PiC) is consistent with the calcium-induced conformational change of PiC in mPTP formation. Co-immunoprecipitation and GST-CyP-D pull-down analysis showed that CYP-D interacts with PiC [39]. The bovine ANT1 of CAT complex is consistent with ANT, which forms MPTP. It also suggests that ANT has a conformation of a large cavity extending from the cytoplasmic side into the membrane, in which a contraction channel comprising three spirals prevents pore formation [40,41]. If conformational changes facilitated by CYP-D rearranged these helices, this may also be responsible for the formation of MPTP.

As a dynamic structure that closes during ischemia and opens during reperfusion, MPTP plays a “switch” role in apoptosis [42,43,44]. MPTP brings destruction to the proton gradient and electric potential of the mitochondrial inner membrane, leads to the inflow of solutes and water, and increases the permeability of mitochondria, thus increasing the burden on the mitochondrial. Subsequent swelling and breakdown of the outer membrane activate the cascade reaction of pro-apoptosis [45,46,47,48]. It can thus be suggested that pro-apoptotic proteins are released into the intracellular mediator, which contributes to the release of cyt c, apoptosis inducing factor (AIF), and the formation of apoptotic bodies [8]. Another important finding was that ATP levels decreased significantly when most hepatocyte mitochondria were affected by MPTP [49]. Recent cases reported by Sun et al. [50] also support the hypothesis that this decline in ATP levels is often accompanied by mitochondria swelling, activation of caspases, up-regulation of Bcl-2, and downregulation of Bcl-2-associated X protein (Bax). Even if a small fraction of MPTP lifts a restriction, mitochondrial homeostasis imbalance and cell apoptosis will still occur [20,51]. Surveys conducted by Cai et al. [52] have shown that the cell apoptosis was effectively offset by inhibition of MPTP opening. During hepatic IRI, MPTP opening is induced by the following alterations: over-production of ROS, calcium (Ca^2+^) overload, and ∆Ψm loss (Table 1).

### 3.1. ROS Triggered MPTP Opening during Hepatic IRI

The early characteristics of hepatic IRI are the occurrence of oxidative stress and the release of ROS, which directly lead to hepatocyte injury [53]. Mitochondria are both targets and generators of ROS [54]. Under normal physiological states, hepatocytes can withstand certain levels of ROS. However, excessive ROS can be produced when damaged mitochondria become dysfunctional, resulting in greater damage to mitochondria [55,56]. Thus far, previous studies have suggested that excessive ROS production during hepatic IRI can result in apoptosis due to the further damage of DNA, proteins, lipids, and other cellular molecules [57].

Evidence suggests that the overproduction of ROS mainly occurs after blood and oxygen return to the hypoxic liver [58]. This increase in ROS allows for mitochondrial permeability transition, depolarization of ΔΨm, and MPTP opening [59]. Another significant aspect of ROS-induced MPTP opening is that ROS interacts with polyunsaturated fatty acids in biofilms to produce highly toxic and reactive molecules, resulting in the production of lipid peroxides and poisonous aldehydes, such as 4-hydroxynonenal (4-HNE) or malondialdehyde (MDA). Firstly, the accumulation of 4-HNE may activate a series of signaling pathways that lead to liver cell apoptosis, including decreased integrity of the mitochondrial membrane, increased permeability of mitochondrial, and inhibited electron transport chain [60,61]. Secondly, MDA is a major metabolite of lipid peroxides that have been demonstrated to be the main cause of cell membrane damage [62].

As key binding targets for carbon monoxide (CO), mitochondria are an important target for CO-dependent regulation of cellular physiology and signal pathways due to the richness in iron such as heme [63]. The toxicity of CO on mitochondria has been broadened to relate to ROS generation, ΔΨm, mitochondrial respiration, and mitochondrial-dependent metabolic pathways. As noted by Jung et al. [64], CO regulates the production of mitochondrial ROS in a specific manner. At high concentrations, CO can inhibit mitochondrial respiration and ATP production, and regulate the glycolytic pathway in a dose-dependent manner [65]. On the other hand, as the key catalyst for the formation of ROS, iron is the main instigator of MPTP. Intracellular chelated iron can promote hepatocyte oxidative damage and MPTP-induced apoptosis [66]. During the phase of ischemic, the lysosome releases chelatable ferrous iron (Fe^2+^), and then Fe^2+^ is absorbed into mitochondria by mitochondrial Ca^2+^ uniporter. Once the Fe^2+^ overload occurs in mitochondria, the formation of hydrogen peroxide after reperfusion leads to the production of hydroxyl radicals (OHD). In particular, OHD will damage DNA, protein, and cell membranes, resulting in MPTP opening and cell apoptosis [67].

### 3.2. Calcium Overload and MPTP Opening during IRI

The imbalance of Ca^2+^ homeostasis is a common way that has a considerable impact on hepatocyte injury. Intracellular Ca^2+^ concentration is approximately 10–100 nM, 10,000 times lower than extracellular Ca^2+^ concentration. This gradient can be maintained by four mechanisms: (1) ATP-mediated transmembrane outflow; (2) Na^+^/K^+^-mediated Ca^2+^/Na^+^ retrograde transport; (3) Ca^2+^ storage capacity of endoplasmic reticulum; and (4) oxygen-dependent intracellular Ca^2+^ pump in mitochondria [68,69]. However, with the increased concentration of cytoplasmic Ca^2+^, mitochondria can act as a buffer for redundant Ca^2+^. This subsequently leads to the migration of abundant intracellular Ca^2+^ due to the increase in intracellular Na^+^ concentration and the antiport of Na^+^/Ca^2+^. Moreover, ischemia and hypoxia lead to an increase in cell membrane permeability, resulting in a further pile-up of the intracellular Ca^2+^. Ischemia and hypoxia also violate the structure and function of mitochondria, resulting in the release of large amounts of Ca^2+^ from the endoplasmic reticulum (ER) and intracellular Ca2^+^ overload [70].

With the development of research, the molecular structure of mitochondrial calcium ion transport has been identified as mitochondrial Ca^2+^ uniporter (MCU), Na^+^/Ca^2+^ exchanger (NCLX) and Ca^2+^/H^+^ antiporter (Letm1) [71]. The consensus is that MCU is primarily responsible for mitochondrial Ca^2+^ influx and that MCU promotes Ca^2+^ transport down its electrochemical gradient. MICU1 is an adjustable MCU containing Ca^2+^ binding EF-Hand structure [72]. When intracellular Ca^2+^ concentration is low, it prevents Ca^2+^ from entering the MCU channel. The MiCU1/MiCU2 ratio, and its interaction with MCU, determine the dynamics of Ca^2+^ transport to mitochondria. Overexpression of MICU1 gene also results in significantly sped up Ca^2+^ entry into mitochondria [72]. Letm1(K^+^/H^+^ exchanger), mediates mitochondrial Ca^2+^ and H^+^ transport. With the dual role of Letm1 as a Ca^2+^/H^+^ exchanger, Letm1 transports Ca^2+^ in and out of mitochondria in a Ca^2+^ and pH gradient-dependent manner [72]. When I/R injury occurs, the level of intracellular ATP decreases, resulting in down-regulation of the activity of ATP-dependent Na^+^/K^+^-AT-pase embedded in the cell membrane [73].

Mitochondria Ca^2+^ overload can promote lipid peroxidation and weaken the oxidative phosphorylation of mitochondria, resulting in impaired structure and function of mitochondria and decreased ATP synthesis rate [69]. Interestingly, the damaged mitochondrial membrane structure caused by lipid peroxidation aggravates Ca^2+^ overload. Therefore, both mitochondria Ca^2+^ overload and lipid peroxidation generally occur in liver damage after IRI. Further analysis showed that the increase in intracellular Ca^2+^ during the ischemia period also promoted the production of XO. Chang et al. [74] conclude that cell reoxygenation in the process of reperfusion causes XO to induce the production of superoxide and the restoration of ATP levels, which allows mitochondria to actively uptake Ca^2+^, resulting in a large amount of Ca^2+^ overload. To maintain the integrity of the mitochondrial membrane, the mitochondrial ATP synthase reverses its activity to provide energy for different ion pumps in the mitochondrial membrane [75]. However, this further increases the inflow of Ca^2+^. Mitochondrial Ca^2+^ overload leads to mitochondrial membrane damage, especially the decrease in mitochondrial transmembrane potential and MPTP opening. As a result, pro-apoptotic factors are released to the cytoplasm and accelerate cell death [76].

The relationship between Ca^2+^ and MPTP opening is complex and related to a variety of different pathways. Ca^2+^-induced MPTP opening has three separable but interrelated mechanisms. Firstly, hypoxia has been reported to be an effective disruptor of oxidative phosphorylation, hindering the production of ATP and leading to Ca^2+^ overload [20]. It was suggested that the extent mitochondrial Na^+^/Ca^2+^ commutator overburdened and inactive state exacerbated with Ca^2+^ overload, causing the concentration of Ca^2+^ to increase enough to trigger the activation of MPTP opening [26,74]. Secondly, the increase in intracellular Ca^2+^ content induces the formation of protein kinase C (PKC). Nakazato et al. [77] points out that PKC shows a strong indirect elevating effect on nuclear transcription factor kappa B (NFkB) activation and ROS production, thus promoting MPTP opening. Lastly, intracellular Ca^2+^ overload can activate Ca^2+^-dependent enzymes, such as calpain, and phospholipase C [78]. Calpain is a Ca^2+^-dependent intracellular cysteine protease [79]. An uncontrolled increase in Ca^2+^ levels can lead to continuous activation of calpain. Cannistra et al. [66] suggested that calpain-induced degradation of autophagy-related 7 and Beclin-1 leads to autophagy defect and MPTP-dependent hepatocyte death after IRI.

### 3.3. Mitochondrial Membrane Potential Loss and MPTP Opening during IRI

ΔΨm is an increasingly important aspect in maintaining mitochondrial function and inhibiting hepatocyte apoptosis [80]. The loss of ΔΨm is one of the earliest events in the cascade of apoptosis. Under normal physiological conditions, the existence of ΔΨm mainly depends on the closed MPTP [5]. In light of recent research, MPTP opening destroys the integrity of the mitochondrial membrane and makes the ΔΨm lose or collapse [74]. After ΔΨm loss, the synthesis of mtRNA and protein was blocked, followed by uncoupling oxidative phosphorylation and ATP depletion, resulting in osmotic swelling and outer membrane rupture, leading to mitochondrial permeability transition, releasing apoptosis-driving factors such as cyt c, which leads to cell apoptosis [81]. 

Ca^2+^/calmodulin dependent protein kinase II (CaMKII) proved an important member of the CaMK family. CaMKII is a kind of protein activated by Ca^2+^ and calmodulin. Zhang et al. [7] demonstrated that CaMKIIγ could induce the change of ΔΨm and mitochondrial permeability. In the same vein, Kang et al. [82] found that the overexpression of CaMKII γ caused significant ultrastructural damage, such as mitochondria swelling, hepatocyte necrosis, mitochondria membrane rupture, and atrophy. Furthermore, phospholipases of CaMKII can regulate the influx of Ca^2+^ into mitochondria [76]. This mechanism is similar to that reported by Joiner et al. [83] who report CaMK II activity can regulate the influx of Ca^2+^ into mitochondria and promote apoptosis.

### 3.4. Regulatory Role of Akt/GSK-3β Pathway on MPTP Opening

The Akt/GSK-3β pathway has significant biological functions in MPTP opening. Many prosurvival signaling pathways inactivate glycogen GSK-3β by regulating phosphorylated GSK-3β, then increase the opening of MPTP and regulate the IRI [84]. The reperfusion injury salvage kinase (RISK) and survivor activating factor enhancement (SAFE) pathways are considered the two main pathways of MPTP opening [85,86]. To date, previous research has shown that GSK-3β at Ser9 is phosphorylated by Akt, resulting in the interaction with MPTP regulatory factors and inhibiting the opening of MPTP during reperfusion [45].

At the same time, the phosphorylation of GSK-3β can also actively regulate β-Catenin [87]. Zhao et al. [84] demonstrate that administration of GSK-3β inhibitors before IRI can increase the accumulation of intracellular β-catenin, thus activating the GSK-3β/β-catenin signaling pathway and further enhancing the expression of Bcl-2.

## 4. The Release of Apoptosis-Related Proteins

During hepatic IRI, apoptosis-related proteins are released from the intermembrane space to the cytoplasm due to the undermined structure of mitochondria. The release of apoptosis-related proteins after IRI is known to activate the apoptosis-related signaling pathway [88]. Thus far, previous studies have demonstrated that the proteins related to apoptosis include cyt c, AIF, b-catenin, caveolin-1, and endonuclease, as well as mitochondria apoptosis proteins such as the second mitochondrial activator of caspase (Smac) and mitochondrial serine protease [89,90]. Among those apoptosis-related proteins, the release of cyt c has tighter relations with apoptosis.

**Figure 3:**
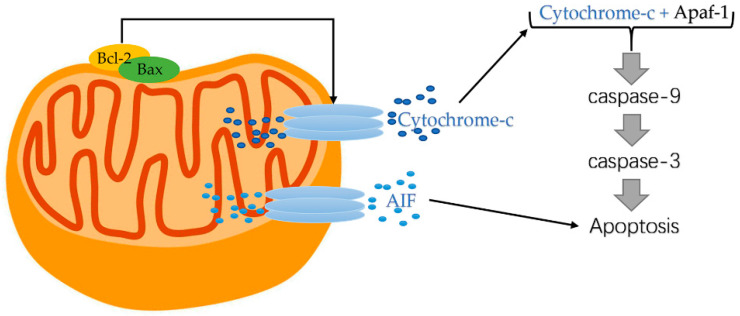
Cytochrome-c released from mitochondrial. Cyt c released from mitochondria enters the cytoplasm, resulting in blocked respiratory chain and electron transport of mitochondria, reduced energy supply of cells, and the conformational changes of mitochondria. Cyt c forms apoptotic bodies and participates in the apoptosis pathway of Caspase cells.

Cyt c function as an apoptosis protein activator due to its electron carrier role in the mitochondrial electron transport chain [91]. Cyt c released from mitochondria enters the cytoplasm, resulting in a blocked respiratory chain and electron transport of mitochondria, reduced energy supply of cells, and the conformational changes of mitochondria [81]. Subsequently, as shown in Figure 3, a polymer composed of apoptotic protease activating factor-1(Apaf-1), cyt c, and Caspase3, namely apoptotic bodies, is formed, which triggers the caspase cascade and leads to apoptosis [46,92]. 

According to Kalpage et al. [92], the phosphorylation of cyt c during IRI aggravates tissue injury through maximum electron transport chain flow, ΔΨm hyperpolarization, and excessive ROS production. The accumulation of oxidized cyt c in the cytoplasm can lead to apoptosis [93,94]. In addition, the adaptor protein p66shc oxidizes cyt c to produce ROS, which leads to MPTP opening, followed by the release of more cyt c into the cytoplasm [95].

Previously published studies on the regulatory mechanism of cyt c release during apoptosis are not fully understood. However, it has been established that Bcl-2, Bcl2-like 1(Bcl-xL), and Bax protein are major regulators of VDAC which may exert certain effects in regulating the release of cyt c [96]. To the start, stimulation of proapoptotic factors resulted in the decreased capability of Bcl-2 in maintaining the integrity of the mitochondrial membrane and enforced Bax translocation outside the mitochondrial membrane. Afterwards, cyt c is released from the mitochondrial matrix owing to mitochondrial permeability transition and decreased mitochondrial transmembrane potential. In the next place, the released cyt c enters the cytoplasm and binds to Apaf-1 to form an oligomer, activating Caspase-9 and downstream Caspase-3 [19].

Cherian et al. [97] demonstrated that the AIF pathway is another typical way of apoptosis. AIF has antioxidant and survival-promoting effects when it is located in mitochondria. However, AIF transferred to the nucleus after being released from the intermembrane space triggering caspase-independent apoptotic via activating poly (ADP-ribose) polymerase-1 (PARP-1) [98].

## 5. Regulatory Role of B Cell Lymphoma-2(Bcl-2) Family Proteins

**Figure 4:**
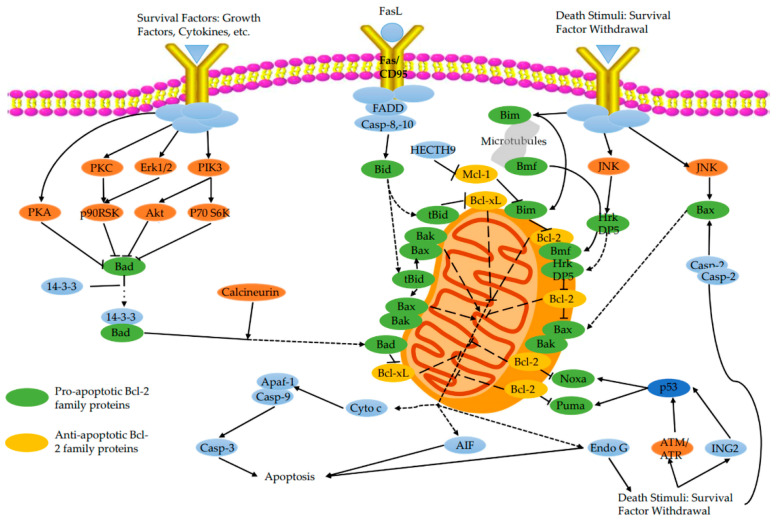
Signal transduction of pro-and anti-apoptotic Bcl-2 family proteins. Bcl-2 family proteins can accurately promote the mitochondrial permeability transition that allows the outflow of mitochondrial content. They can also induce the opening of mitochondrial MPTP and promote the release of apoptosis-related proteins. The Bcl-2/Bax ratio of the Bcl-2 family determines cell survival or apoptosis after certain stimuli.

The Bcl-2 family proteins can be categorized into two groups: (1) anti-apoptotic factors such as Bcl-2 and Bcl-xL; (2) proapoptotic factors such as Bax and Bad (Figure 4). On the one hand, Bcl-2 family proteins can accurately promote the mitochondrial permeability transition that allows the outflow of mitochondrial content. On the other hand, Bcl-2 family proteins can induce the opening of MPTP and promote the release of apoptosis-related proteins [99]. Bax and Bcl-2 are the most widely researched member of Bcl-2 family proteins and have been extensively regarded as a key factor in the activation or inhibition of apoptotic pathways. Pro-apoptotic Bax promotes apoptosis by forming oligomers in the outer membrane of mitochondria and participating in the release of apoptosis-related molecules such as cyt c. By contrast, anti-apoptotic Bcl-2 inhibits mitochondrial apoptosis by blocking the release and oligomerization of Bax [100]. In short, Bax can trigger the release of cyt c to the cytoplasm, while Bcl-2 inhibits cyt c release. 

### 5.1. Pro-Apoptotic Bcl-2 Family Proteins

Under normal physiological conditions, pro-apoptotic Bcl-2 proteins such as Bax, BH3-Interacting domain death agonist (Bid), Bcl2 associated death promoter (Bad), and Bcl-2 interacting mediator (Bim) reside in the cytoplasm. However, these proteins translocate to mitochondria after receiving apoptosis signals and then promote the release of cyt c.

Accordingly, cells under the stress state exhibited translocate of Bax and mitochondrial permeability transition, which promotes the release of cyt c from the intermembrane space to the cytoplasm [101,102]. Furthermore, Bax is involved in the interaction with a series of membrane proteins, such as ANT and VDAC, to enhance the mitochondrial release of cyt c, AIF, and SMAC/DIABLO. The binding of Ca^2+^ to cardiolipin boosts the transport of Bax and Bid from the cytoplasm to the mitochondrial outer membrane [74]. Bid is the only member of the Bcl-2 superfamily that functions to link the extrinsic apoptotic pathway and the mitochondrial amplification loop of the intrinsic pathway [103]. Bid further synergistically enhanced the toxicity of Bax with the increase in mitochondrial Ca^2+^ concentration. As regards Bad, Bad translocates to mitochondria and forms a pre-apoptotic complex with Bcl-xL. Survival factors will inhibit the translocation of Bad, induce Bad phosphorylation, and lead to cytoplasmic aggregation [45]. 

### 5.2. Anti-Apoptotic Bcl-2 Family Proteins

Bcl-2 is the most potent anti-apoptotic protein known. Bcl-2 inhibits apoptosis by inhibiting Bax activity, stabilizing the ΔΨm, and inhibiting cyt c release and caspase activation [104,105,106]. Wu et al. [19] identifies that the overexpression of Bcl-2 prevents the outflow of cyt c from mitochondria and the initiation of apoptosis. By contrast, repressed expression of Bcl-2 leads to Bax protein migration and binding to the permeability transition pore of the mitochondrial membrane, resulting in the loss of selective ion permeability. This gives rise to the release of intermembrane substances such as cyt c and AIF into the cytoplasm [99].

Recent evidence suggests that ROS responded significantly to the balance of anti-apoptotic and pro-apoptotic proteins in mitochondria [107]. As an inhibitor of apoptosis, Bcl-2 can prevent apoptosis caused by free radicals and lipid peroxidation. Bcl-2 has antioxidant properties owing to its participation in the redox process and inhibiting the formation of ROS [104,108]. Aside from reduced IR-induced necrosis and apoptosis, Bcl-2 also reduces ATP production by inhibiting the TCA cycle and mitochondrial respiration [106]. During ischemia, when hypoxia inhibits ΔΨm and mitochondrial ATP production, F1Fo-ATP can reverse and consume the ATP produced by TCA.

## 6. ATP Depletion

Recent research has revealed that the main mechanism of IRI is mitochondrial damage caused by severe ATP depletion in the hepatic microenvironment during ischemia [109]. The ΔΨm is formed by pumping protons through the respiratory chain into the intermembrane space [106]. It is now understood that ΔΨm is a major influencer of the production and destruction of ATP [110]. When cold ischemia occurs, hypoxia and deficiency of metabolites will lead to decreased ATP synthesis, destructed ATP-dependent enzymes, and increased concentration of adenosine diphosphate (ADP). During this period, the ATP produced will be applied to maintain the ΔΨm resulting in insufficient production of ATP [34].

In the case of ATP depletion, cells begin to upregulate survival mechanisms such as protective autophagy to fulfill the energy demand [111], while the ATP depletion touches off the increase in Na^+^, which in turn inhibits the activity of Na^+^/K^+^/ATPase [34]. As ubiquitous enzymes, Na^+^/K^+^/ATPase and Ca^2+^/ATPase are important in maintaining the function of the mitochondrial membrane by regulating the ionic balance [112]. The activity of these ATP enzymes depends to a large extent on the fluidity of the membrane and the level of ATP. When the activity of ATP enzymes is reduced, excessive Na^2+^, Ca^2+^, and hydration will damage the structure of mitochondria [112]. In addition, ATP deficiency leads to the dysfunction of ATP enzyme-mediated ion transport and increases intracellular and mitochondrial Ca^2+^ content, which further aggravates MPTP opening [10]. Mitochondrial uncoupling leads to the decrease in ATP depletion, which plays a decisive role in the pathogenesis of liver diseases. The dysfunctional and uncoupled mitochondria increase the sensitivity of apoptosis. One interesting finding is that the uncoupling protein (UCP) in the mitochondrial inner membrane can eliminate the concentration difference of transmembrane protons on both sides of the mitochondrial inner membrane, slowing down the process of oxidative phosphorylation and hindering the production of ATP [113]. 

## 7. Endoplasmic Reticulum Stress

ER stress is an adaptive response to the accumulation of misfolded proteins in the ER [114]. Thus far, previous studies have indicated that ER stress is closely related to mitochondrial damage. The emergence of ER stress is attributed to many pathological events, such as hypoxia, glucose deficiency, and oxidative stress, as mitochondria have a contact point that establishes a solid connection with the ER [115]. 

**Figure 5:**
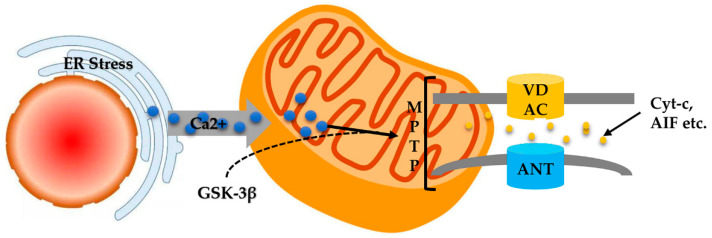
ER stress-induced MPTP opening. ER and mitochondria are firmly connected, ER stress is an adaptive response to the accumulation of misfolded proteins in the ER. With the continuation of ER stress, Ca^2+^ is released from the ER reservoir to the mitochondria, resulting in mitochondrial Ca^2+^ overload, further resulting in MPTP activation and cytochrome C release.

Disturbances of Ca^2+^ homeostasis are another important mediator of ER stress-induced cell apoptosis (Figure 5). With the continuation of ER stress, Ca^2+^ is released from the ER reservoir to the mitochondria, resulting in mitochondrial Ca^2+^ overload [37]. In turn, Ca^2+^ overload induces the synthesis of GSK-3β that activates mitochondrial VDAC, which leads to the activation of MPTP and release of cyt c [116]. Consequently, the pro-apoptotic proteins caspase-9 and caspase-3 were activated [115,117].

## 8. The Role of Nitric Oxide in I/R Injury

Nitric oxide (NO) is a double-edged sword. It participates in the occurrence and development of the pathophysiological process of hepatic I/R injury, regulates local blood flow, inhibits platelet aggregation and leukocyte adhesion, and removes superoxide compounds as an endothelium derived relaxing factor. Superoxide anion is a natural scavenger. However, it also inhibits mitochondrial respiration and reacts with peroxides to produce peroxynitrite, which can cause lipid peroxidation or damage proteins to form nitrotyrosine and induce apoptosis, thus damaging the liver [118]. There are three different nitric oxide synthase (NOS) isoenzymes in organisms, neural nitric oxide synthase (nNOS), inducible nitric oxide synthase (iNOS), and endothelial nitric oxide synthase (eNOS). There are iNOS and eNOS in the liver [119]. Theruvath TP et al. found that eNOS deficiency would aggravate ischemia and reperfusion injury, mainly manifested as increased ALT, necrosis, and apoptosis of liver cells, and increased exudation of mononuclear macrophages in eNOS deficiency group. In addition, blood flow rate and vascular diameter also decreased [120]. Varadarajan R [121] and Hines IN [122] have experimentally confirmed that eNOS-mediated production of NO can alleviate hepatic I/R injury. From these studies, eNOS is a major source of cellular protection through endogenous nitric oxide. Studies have shown that in the early stage of tissue reperfusion, the overproduction of nitric oxide can attenuate the inflammatory cascade and can also be stimulated by the iNOS produced by the pro-inflammatory cascade. The role of NO in ischemia-reperfusion may be: (1) antioxidant free radical activity, reduce the production of lipid peroxidation products, protect the integrity of cell membrane, maintain the normal operation of cell signal transduction system, and protect the enzyme active protein DNA from oxygen free radical attack, blocking the cell apoptosis pathway induced by free radical; (2) protecting the integrity of mitochondrial structure and function, stabilizing the permeability of mitochondrial membrane, preventing calcium overload and apoptosis inducing factor release into the cytoplasm, and preventing the occurrence of apoptosis. It is related to anti-oxygen free radical activity, improving cell energy supply and preventing hepatocyte apoptosis [123].

## 9. Other Mitochondrial-Mediated Apoptosis Signaling Pathways

c-Jun N-terminal kinase (JNK) signaling pathway is one of the considerable pathways that regulate mitochondrial-mediated apoptosis [84]. The causation of the JNK signal pathway activates due to the upregulated level of mitogen-activated protein (MAP) kinases [124]. Zhai C.L. et al. [124] found that JNK may induce apoptosis by regulating the proapoptotic protein Bim. In addition, JNK activation can promote the release of cyt c from mitochondria, cleavage of caspase-3, and necrosis, as well as increase the production of ROS [125].

The Wnt/β-catenin pathway has established its biological role in cell growth and differentiation. Liu et al. [126] demonstrated that the ΔΨm of Wnt1 overexpression mice was stable and that the level of hepatocyte apoptosis involved in the mitochondrial pathway decreased after IRI compared with the control group. Wnt1 overexpression mice also showed an increase in the activity of tricarboxylic acid and the level of ATP [127]. Above all, results indicate that overexpression of Wnt1 can maintain mitochondrial function after IRI.

## 10. Conclusions

Mitochondria have gradually become a popular target for various therapeutic strategies for liver diseases. It is important to discuss the protective mechanism and treatment of hepatic IRI by inhibiting excessive mitochondrial division, improving the mitochondrial fusion barrier [128,129], inhibiting MPTP opening, activating mitochondrial autophagy, and other methods which treat hepatic ischemia reperfusion [130,131]. It was found that gastroditin preconditioning induced autophagy during I/R through ampK-Mammalian target of Rapamycin (mTOR) signaling pathway, including increased pAMPK/AMPK ratio. The p-MTOR/mTOR ratio was decreased, lc3-II expression was down-regulated, and P62 expression was increased [132]. Competition between autophagy and apoptosis may be an important factor in gastrodin reducing apoptosis. Pgc-1 α is the “molecular switch” of mitochondrial biogenesis. Currently, most of the therapeutic drugs studied focus on the activation of PGC-1α and its target genes in order to activate mitochondrial biogenesis and correct the energy crisis under the background of IRI. Pgc-1 α inhibits mitochondrial division by directly and negatively regulating Drp1 expression by binding to the Drp1 promoter. 

The main therapeutic strategies for hepatic IRI include drug therapy, mitochondrial transplantation, and cell or organ transplantation. Drug therapy mainly includes small molecule drugs, natural compound drugs, or protein drugs. Kon et al. [133] reported a novel mitochondrial permeability conversion inhibitor DS44170716, which inhibits Ca^2+^ induced mitochondrial swelling in isolated rat liver by reducing mitochondrial membrane potential, blocking Ca^2+^ entry into mitochondria and inhibiting the activation of MPTP. HepG2 cells were protected from Ca^2+^ induced cell death. Natural compounds such as paeoniflorin [134], quercetin [135], irisin [4], and betulin [136] have been confirmed to prevent or treat liver injury through mitochondrial protection. Lin et al. [137] proved that, in a rat model of hepatic IRI, in vitro mitochondrial transplantation can reduce mitochondrial oxidative stress, cytochrome C release, and liver cell necrosis through overall mitochondrial repair. As a new therapeutic strategy, mitochondrial transplantation is attracting the attention of researchers from various disciplines. However, due to the instability of mitochondria and other factors, implementing this strategy needs a lot of research. Mesenchymal stem cell therapy has proven to be an effective therapy for rapid restoration of mitochondrial function, especially for severe injury caused by liver failure or IRI. Zheng et al. [138] found that bone marrow mesenchymal stem cells can reduce the excessive production of mitochondrial ROS, reduce the accumulation of mitochondrial fragments, restore ATP production and up-regulate mitochondrial mitosis, and improve the apoptosis of liver cells in hepatic IRI.

Combined with the above, the mitochondrial pathway has a pivotal role in apoptosis during hepatic IRI. The mitochondrial pathways emerged as a reliable regulator of apoptosis and provided a theoretical basis for clinical treatment and prevention of hepatic IRI. However, more clinical and laboratory evidence on mitochondrial pathway-mediated apoptosis is still needed to establish a greater degree of accuracy on this matter.

## Figures and Tables

**Table 1:** Influencing factors of MPTP opening. Excessive ROS, elevated calcium, and decreased ΔΨm; these factors are interconnected and can lead to the same events: the MPT pore opening, damage to mitochondrial membranes, and release of pro-apoptotic proteins.

Factors	How	Effects	Results
ROS	excessive ROS produce after reperfusion and CO exposure	promote mitochondrial permeability transition and depolarizes ΔΨm; produce lipid peroxides and other toxic aldehydes	induce MPTP opening
Ca^2+^	Na^+^/Ca^2+^ commutator overburden leads to Ca^2+^ overload	induce PKC formation activate NFkB activate Ca^2+^-dependent enzymes; cause mitochondrial integrity impairment	mitochondrial membrane damage
ΔΨm	mitochondrial integrity impaired causes the ΔΨm loss	block the synthesis of mitochondrial RNA and protein, uncoupling oxidative phosphorylation, and ATP depletion	release of apoptosis drivers, cyt c

## Data Availability

Not applicable.
